# IgE and high‐affinity IgE receptor in chronic inducible urticaria, pathogenic, and management relevance

**DOI:** 10.1002/clt2.12117

**Published:** 2022-02-01

**Authors:** Ana M. Giménez‐Arnau, Clara Ribas‐Llauradó, Nasser Mohammad‐Porras, Gustavo Deza, Ramón M. Pujol, Ramón Gimeno

**Affiliations:** ^1^ Department of Dermatology Hospital del Mar‐Institut d’Investigacions Mèdiques (IMIM) Universitat Pompeu Fabra de Barcelona Barcelona Spain; ^2^ Department of Immunology Hospital del Mar‐Institut Mar d’Investigacions Mèdiques Barcelona Spain

**Keywords:** basophils, chronic inducible urticaria, FcεRI receptor, IgE, omalizumab

## Abstract

**Background:**

IgE and high‐affinity IgE receptor (FcεRI) expression on basophils have been scarcely explored in patients with chronic inducible urticaria (CIndU).

**Objectives:**

To investigate baseline serum IgE and FcεRI expression on blood basophils in a large cohort of CIndU patients and its relationship to treatment response.

**Methods:**

Baseline total serum IgE and basophil FcεRI expression measured by flow cytometry in 165 patients with CIndU was studied. The relationship of both parameters with the response to antihistamine and anti‐IgE (omalizumab) treatment was considered in a subsample of CIndU patients. FcεRI expression in basophils was assessed by mean fluorescence intensity (MFI) and basophil FcεRI standardized density (receptors/cell).

**Results:**

The median FcεRI expression standardized per density in blood basophils was found significantly higher in patients with CIndU compared to HCs. A positive correlation was found between IgE serum levels and basophil FcεRI expression. Basal FcεRI expression was not related to antihistamine treatment response. However, it was related to omalizumab, and patients responding to omalizumab showed higher basal basophil expression of FcεRI levels. Non‐responders to the antihistamine showed significantly higher IgE serum levels.

**Conclusions:**

FcεRI receptor overexpression in patients with CIndU shows almost the same pattern than chronic spontaneous urticaria. It seems to be independent of CIndU subtypes. Although additional studies would be welcome, our work highlights the relevance of FcεRI receptor regulation in CIndU supporting autoimmune basophil and mast cell activation and may be a biomarker for response to anti‐IgE therapy.

## 1 INTRODUCTION

Among the two types of immunoglobulin E (IgE) receptors,^1^, ^2^ low (FcεRII) and high‐affinity (FcεRI) IgE receptors, the latter is constitutively and primarily expressed on mast cells and basophils, where it binds the Fc region of IgE, an immunoglobulin isotype implicated in hypersensitivity and inflammatory and allergic processes.^3^, ^4^, ^5^, ^6^, ^7^

Total IgE serum levels are usually slightly elevated in patients with chronic urticaria (CU) and has been described associated with different response to anti‐IgE therapy.^8^ In this regard, basophil FcεRI expression has already been characterized in chronic spontaneous urticaria (CSU) and it was assessed related to the treatment outcome with anti‐histamine and anti‐IgE therapy.^9^, ^10^, ^11^

The classification of CU into spontaneous and inducible subtypes, and its further subdivision into other subtypes,^12^, ^13^ highlights the need to expand the field's knowledge of CU subtype specificity and differentiation. These phenotypic features of CU may influence therapeutic and treatment decisions, despite seemingly common clinical expression in some cases or overlapping features.^14^ Therefore, a separate study is needed in CIndU as the phenotypic element may be affecting patient's characteristics and, feasibly, the role of IgE in the different diseases.

CIndU is a common inflammatory skin condition characterized by the recurrence of itchy wheals and/or angioedema lasting more than 6 weeks and induced by physical or environmental triggers.^12^, ^15^, ^16^ CIndUs can occur alone or in combination with other types of CIndUs or CSU. The worldwide prevalence of CIndU is not negligible. Even pediatric population shows an estimated CIndU prevalence around 6%, 3% and some studies describe a 17% (26/153) of elderly CU patients that develop an active CIndU.^17^ The prevalence of CIndUs has increased over time^18^ and they have a considerable impact on quality of life.^19^

Overall, the pathophysiology of CU involves activation and degranulation of effector cells, mainly skin mast cells, among others. FcεRI expression is understood to play a key role on these cells in patients with CSU.^9^ However, despite its putative importance in the pathogenesis of all CU forms, to date there are no studies focused on specifically characterizing the role of IgE serum and basophil FcεRI receptor expression in CIndU.

Thus, the main purpose of this preliminary study is to determine the role of IgE serum levels and FcεRI expression on basophils in a large sample of patients with pure or exclusive CIndU. Furthermore, taking into account the effect of omalizumab on FcεRI expression in CSU^9^, ^20^ and that clinical response to omalizumab could be predicted by baseline IgE levels in CSU^21^ but also in CIndU,^22^ in the present study we also assessed whether there are differences in the basal expression of basophil FcεRI receptor in patients with pure CIndU according to whether or not they responded to antihistamines or omalizumab.

## 2 PATIENTS AND METHODS

### 2.1 Participants and study design

This study prospectively included 165 patients with pure CIndU referred to the Urticaria Clinic of the Dermatology Department at Hospital del Mar (Barcelona, Spain) during the period from April 2015 to January 2021. Following a systematized clinical protocol, a complete and structured history (including age, sex, personal clinical history [i.e., atopic diseases including allergic asthma and rhinoconjuntivitis, dermatitis or food allergy, as well as the presence of angioedema]), and laboratory analyses (including total IgE serum levels, blood basophils, or antithyroid antibodies [ATAs] levels, among others) were retrospectively obtained for all patients at their initial medical evaluation. CIndU diagnosis was based on patients' clinical history and the results of standardized provocation testing.^16^ The classification of CIndU subtypes was also characterized. Part of this sample has already been included elsewhere.^11^

As the main aim of the present study, peripheral blood samples from patients with CIndU were analyzed to measure basal total IgE in serum and FcεRI expression in basophils by flow cytometry. To avoid potential interferences, patients who were under treatment with biological therapies, oral corticosteroids, and/or other immunosuppressive agents were excluded from the study.

In addition, peripheral blood samples from a control group of 34 sex‐equivalent healthy adult controls (HCs) with no family or personal history of allergic asthma, allergic rhinitis, CU and atopic dermatitis were included for reference data.

Complementary tests included in the study belong to the normal clinical practice in our daily medical activity. Ethical approval for the study was granted by the local Clinical Research Ethics Committee (ethics approval #2012/4913/I).

### 2.2 Basophil cell preparation and flow cytometry for the high‐affinity IgE receptor (FcεRI) expression

We followed standard procedures to perform flow cytometry analyses on both patients and HCs.^9^ FcεRI expression in basophils was assessed by mean fluorescence intensity (MFI) and also by density of receptors per cell using standard beads (QuantumTM Simply Cellular, Bang Laboratories, Inc.) according to the manufacturer's instructions.

First, 150 μL of anticoagulated blood was incubated on the same day of collection for 20 min at 4°C with an excess of human immunoglobulins to block nonspecific binding. The blood was then stained with anti‐CD123‐PE (clone 9F5, BD Biosciences) and anti‐CD193‐APC (clone 5E8, Biolegend) to identify basophils and with antiFcεR1a‐FITC (clone CRA1; eBioscience) or an isotype control to establish FcεRI expression on the surface of blood basophils.

Samples were lysed and fixed using FACS Lysing Solution (BD Biosciences) and analyzed by flow cytometry in a FACSCanto II using the FACSDiva software. At least 2 × 10^5^ events were acquired.

Basophil FcεRI receptor levels were expressed as MFI. In addition, a standardized and more stable FceRI density measure (receptors/cell) was obtained since the MFI measure can be affected by different parameters.^23^ This standardized quantitative flow cytometry analysis showing the number of receptors per cell was developed based on MFI. The basis of this approach is the calibration of the fluorescence axis and the number of fluorochrome molecules bound to the cell, or directly the antibody binding capacity using standard beads.

To ensure consistency of analysis, the same investigator processed and analyzed all samples.

### 2.3 IgE in serum and antithyroid antibodies (ATAs) levels

Total IgE and circulating antithyroid antibodies (ATAs) levels in serum were analyzed by a chemiluminescence immunoassay technique using the IMMULITE 2000 XPi System (Siemens).

### 2.4 Urticaria control test (UCT) and provocation testing

The total score of the urticaria control test (UCT)^24^, ^25^ was obtained from patients to evaluate treatment outcome. UCT is a validated simple 4‐item questionnaire which can be used for CSU and CIndU and asks patients to retrospectively score the impact of urticaria symptoms on morbidity, quality of life and quality of treatment over the previous 4 weeks; scores <12 on the UCT are indicative of lack of disease control (UCT score of 0: worst possible disease control; score of 16: complete disease control^24^). In addition, thresholds were assessed, when possible, in symptomatic dermographism by the FricTest® 4.0^26^ or acquired cold urticaria. Critical Stimulation Time Threshold (CSTT) and Critical Temperature Threshold (CTT) scores were collected from a cold provocation test with TempTest® 3.0. (EMO Systems GmbH).^27^, ^28^ With this, baseline provocation thresholds were evaluated.

### 2.5 Treatment management

Treatments were applied following the EAACI/GA^2^LEN/EDF/WAO guideline for the management of urticaria.^25^ The percentage of patients responding to antihistamine at licensed and fourfold up‐dosing dose as well as to anti‐IgE therapy, omalizumab, was evaluated. Patients who obtained after 4 weeks with antihistamine and 6 months with omalizumab an UCT ≥12 are considered responders if it was not the case patients were considered not responders. Baseline FcεRI expression and IgE serum levels were evaluated in both groups of patients according to their therapeutics response.

### 2.6 Statistical analysis

All measures were baseline determinations. Descriptive statistics were performed for each variable, using median and range for quantitative variables, and absolute (*n*) and relative (%) frequencies for categorical variables. To compare quantitative and qualitative variables between patients with CIndU and HCs, the Mann–Whitney *U* test and chi‐square test were used, respectively. Likewise, the Mann–Whitney *U* test was used to compare basal FcεRI expression and IgE levels attending to the response to treatments (anti‐histamine therapy and omalizumab). Exploratorily, we also compared UCT and CSTT and CTT scores from the cold provocation test when exploring basal FcεRI expression and IgE levels according to treatment response, in order to clarify outcomes related to the treatment response/nonresponse groups.

Spearman's Rho (*r*_*s*_) correlation was used to assess the association of FcεRI receptor expression with IgE serum levels in the whole CindU sample and by subgroups according to treatment response.

Complementary, exploratory, and post‐hoc analyses were performed to compare the main variables between CIndU subtypes using Dunn's nonparametric multiple comparisons test.

The loss of sample size (*N*) for each variable is shown throughout the results. All analyses were performed with Prism 8.0 software (GraphPad) and a *p* < 0.05 was considered statistically significant.

## 3 RESULTS

### 3.1 Sociodemographic and clinical characteristics of the sample

A final sample of 165 patients with pure CIndU was referred to our clinic and were therefore included in the present analysis. The demographic, clinical and laboratory characteristics of the study population are summarized in Table 1. The diagnostic distribution of CIndU subtypes was as follows: 72 patients presented with cold urticaria, 35 with symptomatic dermographism, 26 with solar urticaria, 24 with cholinergic urticaria, and 8 with delayed pressure urticaria (see Table 2).

**TABLE 1:** Demographic, clinical, and laboratory characteristics of the entire sample

	Chronic inducible urticaria (*N* = 165)	Controls (*N* = 34)	Statistical test
Sociodemographic data			
Sex (female)	102 (61.82%)	21 (61.76%)	3.415^e^ ^−005^ (*p* = 0.995)
Age (years)	39 (3–81)	49 (15–79)	1914 (*p* = 0.003)
Personal clinical history			
Atopy	20 (12.12%)	0 (0%)	4582 (*p* = 0.032)
Angioedema	12 (7.27%)	0 (0%)	
Thyroid diseases	12 (8.51%)^a^	0 (0%)	
Laboratory analyses			
Antithyroid antibodies (ATAs)	13 (9.29%)^b^	NND	
Total baseline IgE serum levels, IU/mL	105 (2.6–10,603)^c^	ND	
Blood basophil count, ×103 µ/mcL	0.04 (0–0.14)	ND	
FcεRI expression on basophils, MFI	11,891 (603–40,139)^c^	9201 (2043–14,710)	2049 (*p* = 0.0492)
FcεRI expression on basophils, receptors/cell (density)	482,377 (11,774–937,007)^d^	226,234 (33,838–861,009)	720 (*p* < 0.0001)
Treatment response^e^			
Response to a H1 antihistamine	121 (73.78%)^f^	Na	
No response to H1 antihistamine	43 (26.22%)	Na	
Response to omalizumab	34 (89.47%)^g^	Na	
No response to omalizumab	4 (10.53%)	Na	

*Note*: *N* (%) or median (range). Mann–Whitney *U* test used for quantitative variables and chi‐square test used for qualitative variables.

Abbreviations: antiH, antihistamine; MFI, mean fluorescence intensity; Na, not applicable; ND, not determined; UCT, urticaria control test.

^a^ *n* = on a sample of 141.

^b^ *n* = on a sample of 140.

^c^ *n* = on a sample of 153; *n* = on a sample of 62.

^d^ *n* = on a sample of 84.

^e^Scores <12 on the UCT are indicative of lack of disease control. Scores ≥12 on the UCT are indicative of disease control.

^f^ *n* = on a sample of 164.

^g^ *n* = on a sample of 38.

**TABLE 2:** Demographic, clinical, and laboratory characteristics of the entire sample by CIndU subtypes

	Cold urticaria (*N* = 72)	Solar (*N* = 26)	Cholinergic (*N* = 24)	Dermographism (*N* = 35)	Delayed pressure (*N* = 8)
Sociodemographic data					
Sex (female)	49 (68.06%)	17 (65.38%)	14 (58.33%)	18 (51.43%)	4 (50%)
Age (years)	39 (3–79)	41 (11–77)	32 (9–66)	43 (10–81)	46.5 (23–76)
Personal clinical history					
Atopy	4 (14.81%)	4 (15.38%)	4 (16.67%)	7 (20%)	1 (12.5%)
Angioedema	7 (25.93%)	0	1 (4.17%)	2 (5.71%)	2 (25%)
Thyroid diseases	1 (1.96%)	2 (8.70%)	2 (8.33%)	6 (17.14%)	1 (12.5%)
Laboratory analyses					
Antithyroid antibodies (ATAs)	1 (1.96%)	3 (13.04%)	3 (12.5%)	3 (8.57%)	0
Total baseline IgE serum levels, IU/mL	87.75 (2.6–6678)	205 (21.9–10,606)	72.2 (4.22–3892)	124 (2.64–2530)	81.55 (21.5–304)
Blood basophil count, ×10^3^ µ/mcL	0.04 (0.01–0.1)	0.035 (0.02–0.07)	0.05 (0.02–0.14)	0.05 (0.02–0.1)	0.01
FcεRI expression basophils	10,387 (923–29,807)	16,020 (603–38,828)	12,375 (982–33,547)	13,098 (3200–40,741)	13,294 (7715–16,108)
FcεRI expression basophils, receptors/cell	472,834 (32,748–875,013)	510,809 (227,182–856,116)	384,615 (38,142–355,715)	512,669 (49,577–937,007)	231,723 (214,414–249,032)
Treatment response^a^					
Response to anH1 antihistamine	50 (69.44%)	19 (73.08%)	21 (87.5%)	27 (79.41%)	4 (50%)
No response to H1 antihistamine UCT<12	22 (30.56%)	7 (26.92%)	3 (12.50%)	7 (20.59%)	4 (50%)
Response to omalizumab	15 (78.95%)^b^	7 (100%)	3 (100%)	7 (100%)	2 (100%)
No response to omalizumab UCT <12	4 (21.05%)	0 (0%)	0 (0%)	0 (0%)	0 (0%)

*Note*: *N* (%) or median (range).

Abbreviations: antiH, antihistamine; MFI, mean fluorescence intensity.

^a^ Scores <12 on the UCT are indicative of lack of disease control. Scores >12 on the UCT are indicative of disease control.

^b^ *n* = on a sample of 19.

### 3.2 Basal FcεRI basophil expression and IgE serum levels in patients with CIndU

**FIGURE 1:**
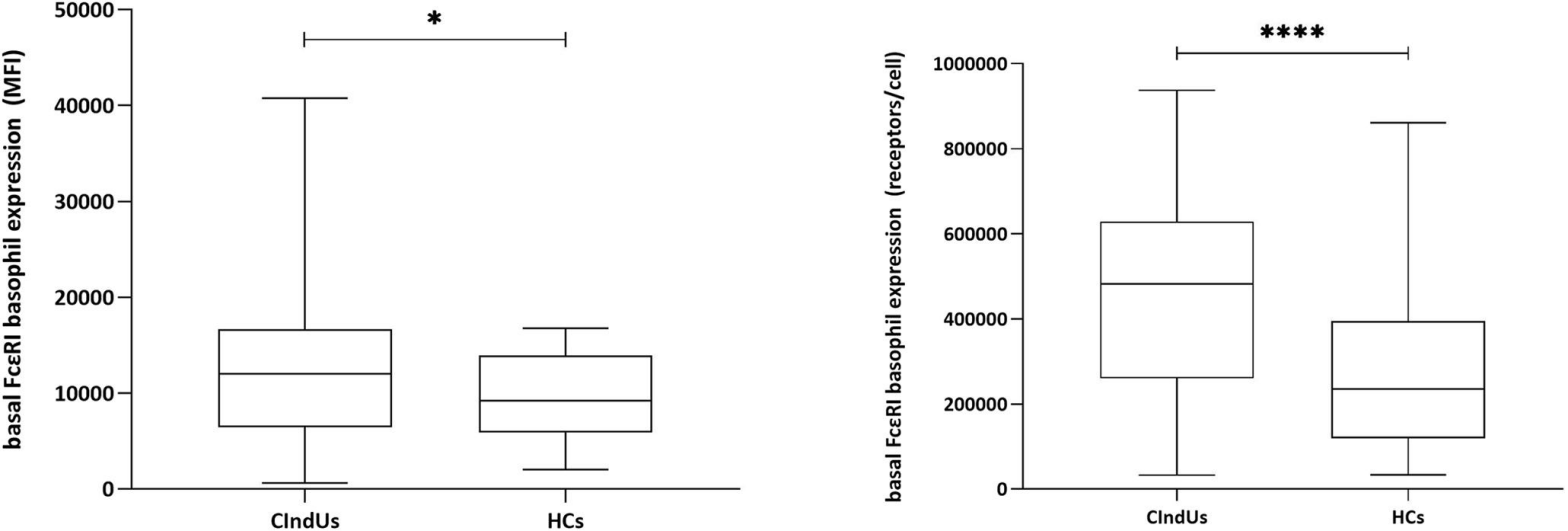
Differences between patients with CIndU and healthy controls in basal basophil expression of the FcεRI receptor. *p < 0.05; ****p < 0.0001

FcεRI expression in basophils differed significantly between patients with CIndU and HCs, especially when assessed through receptor density (CIndU: median, 482,377; HCs: 226,234; *U*[*n*1 = 83, *n*2 = 34] 720, *p* < 0.0001; MFI: CIndU: median, 11,891, HCs: 9201; *U*[*n*1 = 153, *n*2 = 34] 2040, *p* = 0.0492; Figure 1). IgE serum levels in patients with CIndU are shown in Table 1.

**FIGURE 2:**
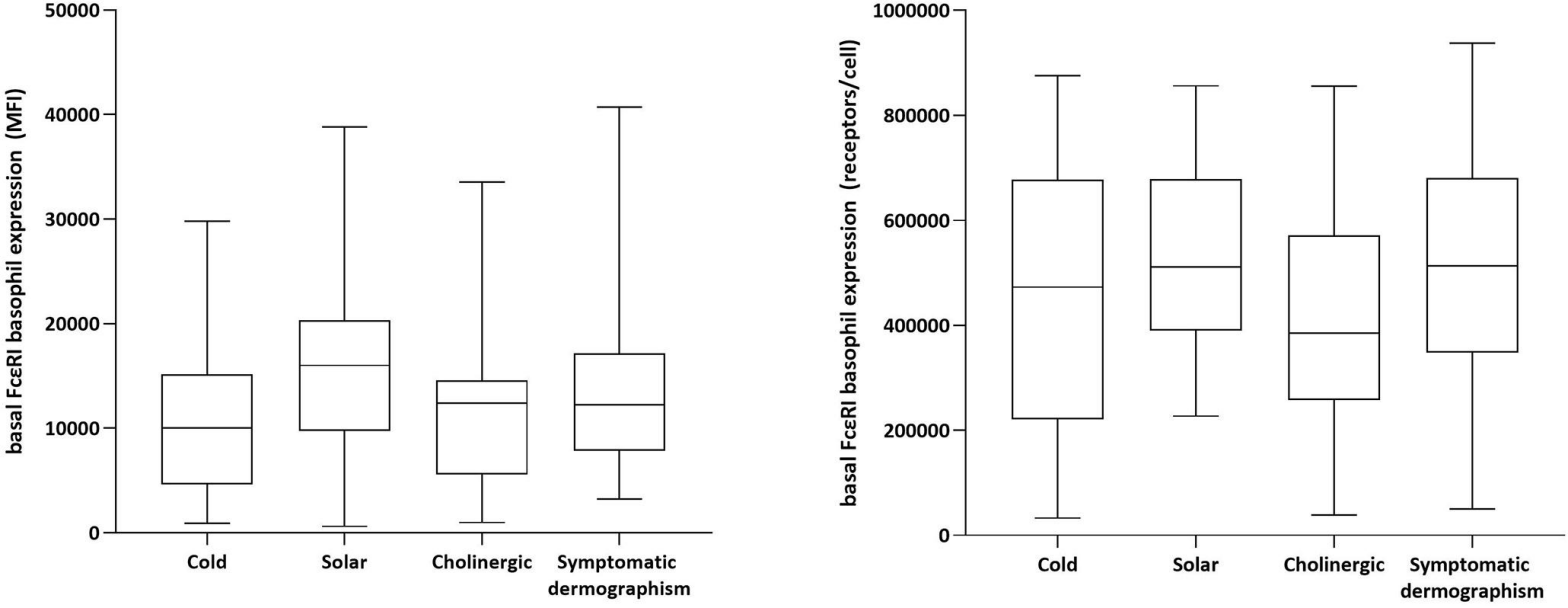
Basal basophil expression of the FcεRI receptor according to CIndU subtypes: cold urticaria, solar urticaria, cholinergic urticaria and symptomatic dermographism. The group with delayed pressure urticaria is not plotted in the figure because only eight cases are reported for the MFI variable, while only two cases are reported for the density variable

Complementary analysis comparing FcεRI expression between CIndU subtypes showed no significant differences between subgroups in either MFI values (*p* > 0.08 in all cases) or density values (*p* > 0.8 in all cases). In this vein, there were no significant differences in total IgE serum levels between CIndU subtypes (*p* > 0.3 in all cases; Table 2 and Figure 2).

### 3.3 Correlation between basal basophil expression of the FcεRI and total IgE in patients with CIndU

**FIGURE 3:**
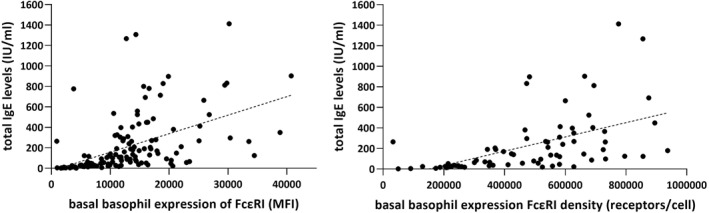
Correlation between basal basophil expression of the FcεRI receptor and total IgE in patients with CIndU. For illustrative purposes the figure does not show the outliers. Nevertheless, the results remain significant after removing the outliers

For the entire CIndU sample, we observed a positive and significant correlation between total IgE levels and both our MFI measure (*r*_*s*_ = 0.708, *p* < 0.0001) and density measure (*r*_*s*_ = 0.708, *p* < 0.0001), indicating that higher basal basophil expression of FcεRI levels was associated with higher IgE serum levels in patients with CIndU (Figure 3). These results remained significant when assessing CIndU subtypes (*r*_*s*_ > 0.426, *p* < 0.023 in all cases).

### 3.4 Therapeutic response and expression of the basophil FcεRI receptor and the IgE levels

#### 3.4.1 Antihistamine treatment

A total of 73.8% responded to antihistamine therapy while the remaining 26.2% did not respond (see Table 1).

**FIGURE 4:**
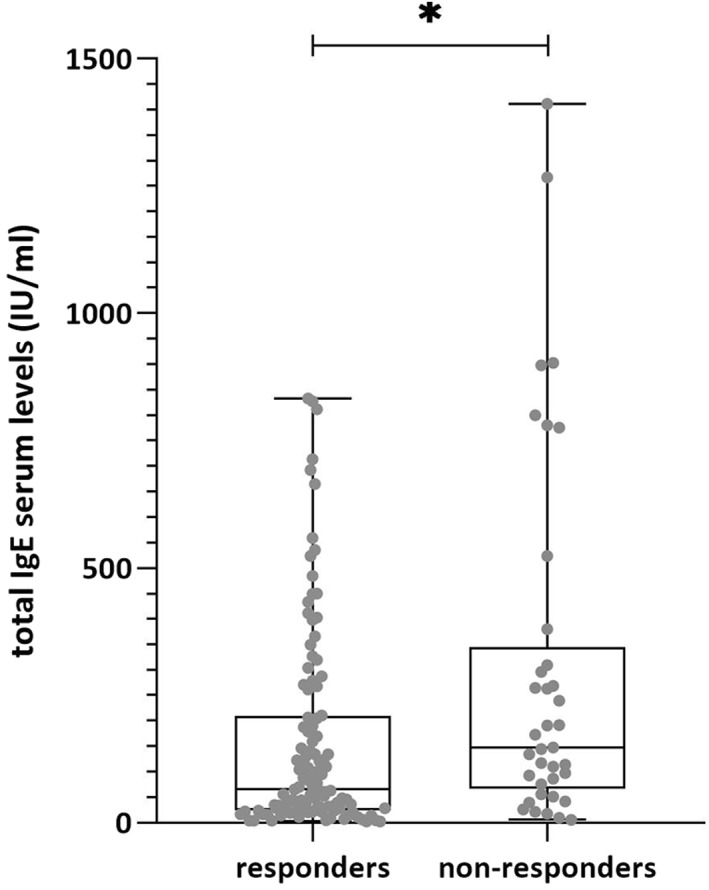
Total IgE serum levels between the responders and non‐responders to H1 antihistamine therapy. For illustrative purposes the figure does not show the outliers. Nevertheless, the results remain significant after removing the outliers. *p < 0.05

There were no significant differences in baseline FcεRI expression between responders and non‐responders to antihistamine therapy according to MFI (responders: median, 11,678; non‐responders: 13,091; *U*[*n*1 = 111, *n*2 = 42] 2047, *p* = 0.2471) or to receptor density (responders: median, 420,346; non‐responders: 586,426; *U*[*n*1 = 62 *n*2 = 20] 459, *p* = 0.0831). However, we found statistically significant differences in total IgE serum levels between the responders and non‐responders to antihistamine therapy (responders: median, 75; non‐responders: 160.5; *U*[*n*1 = 114, *n*2 = 38] 1596, *p* = 0.0149), and with non‐responders to antihistamine showing higher IgE serum levels (Figure 4).

We observed a significant positive correlation between basal FcεRI expression by both MFI and receptor density and IgE serum levels in antihistamine responders (MFI: *r*_*s*_ = 0.7668, *p* < 0.0001); receptor density: *r*_*s*_ = 0.8111, *p* < 0.0001), and in the non‐responders only for MFI values (MFI: *r*_*s*_ = 0.4822, *p* = 0.0025; receptor density: *r*_*s*_ = 0.3970, *p* = 0.0831; in this case 17 pairs were missing). The results in the respondent patients were maintained when evaluated by CIndU subtypes (*r*_*s*_ > 0.400, *p* > 0.0482 in all cases). However, the results were not significant in non‐responders when evaluated by CIndU subtypes (*r*_*s*_ **> ±0.1667, *p* > 0.0630 in all cases).

Complementarily, comparison of CSTT and CTT values between responders and non‐responders to antihistamines showed a significant difference between subgroups in both CSTT (responders: median, 3.5; non‐responders: 1; *U*[*n*1 = 34, *n*2 = 17] 118, *p* = 0.0004), and in CTT (responders: median, 14; non‐responders: 18; *U*[*n*1 = 35, *n*2 = 17] 169, *p* = 0.0108), with non‐responders to treatment showing a provocation threshold appearing in shorter time and at higher temperature, respectively, which would be indicative of a more severe disease.

#### 3.4.2 Omalizumab

Evaluation of response to omalizumab of patients suffering from CIndUs showed that 89.5% responded to treatment and that the remaining 10.53% did not respond (see Table 1). About 20 cold urticaria, 9 solar urticaria, 3 cholinergic urticaria, and 6 symptomatic dermographism were treated with omalizumab. Of these, 14 cold urticaria, 7 solar urticaria, 3 cholinergic urticaria and 2 symptomatic dermographism reached UCT 16 after the sixth administration of omalizumab 300 mg.

There were significant differences in baseline FcεRI expression between responders and non‐responders to omalizumab by MFI (responders: median, 14,228; non‐responders: 3163; *U*[*n*1 = 34, *n*2 = 4] 7, *p* = 0.0012), with omalizumab responders showing higher baseline expression of FcεRI levels. We found no statistically significant differences between omalizumab responders and non‐responders in total IgE serum levels (responders: median, 169.5; non‐responders: 446.5; *U*[*n*1 = 30, *n*2 = 2] 24, *p* = 0.6815). Nevertheless, the four patients with unresponsive acquired cold urticaria showed total baseline IgE levels of below 40 UI/ml.

Complementarily, comparison of CSTT and CTT values between responders and non‐responders to omalizumab showed no significant differences between either subgroup, in CSTT (responders: median, 1; non‐responders: 0.75; *U*[*n*1 = 10, *n*2 = 4] 17, *p* = 0.7253) nor in CTT (responders: median, 20; non‐responders: 17; *U*[*n*1 = 10, *n*2 = 4] 16.5, *p* = 0.6563).

Finally, examination of CIndU patients partially responding to omalizumab (UCT ≥12 < 16) or fully responding (UCT = 16) showed that there were no significant differences in baseline FcεRI expression by MFI (partial responders: median, 13,711; full responders: 13,297; *U*[*n*1 = 20, *n*2 = 12] 120, *p* > 0.999), receptor density (partial responders: median, 581,667; full responders: 682,218; *U*[*n*1 = 11, *n*2 = 6] 19, *p* = 0.1802) or in total serum IgE levels (partial responders: median, 169.5; full responders: 145.0; *U*[*n*1 = 18, *n*2 = 9] 75, *p* = 0.7814).

## 4 DISCUSSION

This is the first study evaluating basal FcεRI receptor characteristics in patients with CIndU. Our data showed that there were differences trending on significance between CIndU patients and HCs in FcεRI levels in basophils by MFI, which were highly significant according to receptor density results. This was independent of CIndU subtypes.

### 4.1 High‐affinity IgE receptor (FcεRI) basal expression

The results of this study in patients with CIndU are in line with those reported for CSU samples, with patients with CIndU presenting higher basal FcεRI receptor levels than HCs.^9^ In a complementary study we have compared these results with those from a cohort of CSU patients (*N* = 79) and there were no differences in basal receptor levels between patients belonging to these two groups (MFI: *p* = 0.4118; receptor density: *p* = 0.0844).

### 4.2 Correlation between basal expression of the FcεRI and total IgE serum levels

In this vein, patients with CIndU presented a positive association between basal expression of FcεRI receptor and IgE serum levels. IgE thresholds may vary between laboratories, but IgE levels of <100 or >100 IU/ml are often considered normal or extremely likely for allergic risk symptoms, respectively.^29^ Interestingly, in our sample, median IgE levels were close to normal (105 IU/ml), although the range was very wide. While there is more evidence of slightly elevated IgE levels in CSU,^30^ there is little data on CIndU. In this sense, it should be noted that a low IgE level may not always indicate absence of active urticaria.^30^

### 4.3 Therapeutic response and expression of the basophil FcεRI receptor and the IgE levels

#### 4.3.1 Antihistamine treatment

Data on basal FcεRI receptor and IgE serum levels according to the treatment approach may provide more information in this regard. As it was suggested, in those who do not respond to antihistamine therapy, and who will therefore require treatment with omalizumab, we found higher total IgE serum levels versus responders to antiH1. In addition, in some cases (chronic acquired cold urticaria) the provocation test found greater disease severity in non‐responders, which does not happen when we compare patients who are responders and non‐responders to omalizumab. However, there were no significant differences in baseline FcεRI expression between responders and non‐responders to antihistamine therapy although both showed higher FcεRI expression than HCs. As such, the data on the FcεRI expression per se does not serve as a predictive biomarker of response to antihistamines, as shown in CSU,^11^ while the IgE serum data does shed more light on the antihistamine response, although the current median for the entire sample is not highly important.

As we know that higher IgE means higher expression of FceRI in blood basophils in CIndUs. This suggest that the main mast cell and basophil activating factor in CIndUs through FcεRI is as in CSU an autoimmune mechanism. This autoimmune mechanism would be driven by precise today unidentified auto allergen that are only activated under certain circumstances induced by low temperature, ultraviolet or visible light, sweat and others. We can hypothesize that elevated total IgE at baseline would activate FceRI inducing urticaria, so omalizumab *might help control patients with elevated total IgE at baseline more than antH1*.

#### 4.3.2 Omalizumab

For adults, there is good evidence that omalizumab is efficacious in CSU, with it having a notable effect on basophil FcεRI receptor density.^9^, ^10^, ^11^, ^31^ Clinical evidence in CIndU is not as extensive, and based on small samples, but it is increasing.^32^, ^33^, ^34^ The current literature is slightly contradictory, with some articles postulating that omalizumab shows less efficacy in CIndU compared to CSU, but this has not yet been addressed systematically,^34^ while others support the use of omalizumab in the treatment of patients with antihistamine treatment‐resistant CIndU.^33^ Contrary to what was observed when differentiating patients according to the antihistaminic response, total IgE serum levels and disease severity did not show significant differences according to the response to omalizumab in patients with CIndU, though higher baseline FcεRI levels in responders to omalizumab versus non‐responders was observed. Previous studies in CU in general^11^ and in CSU in particular^9^ advocate basophil FcεRI expression as a potential immunological predictor of response to omalizumab therapy. Our data corroborate in patients with pure CIndU what previous studies in other urticaria samples have shown, which is that patients who respond to omalizumab have higher basal expression levels of the FcεRI receptor in basophils than non‐responders. In addition, non‐responders even show a lower FcεRI median than HCs. Thus, basal levels of the FcεRI receptor would also appear as a biomarker for predicting response to anti‐IgE treatment in CIndU.

With regards to total IgE serum levels, although the median in non‐responders to omalizumab is well above what is considered the threshold for allergic response (>400 IU/ml) and what appears to be far removed from those presented by the respondents to omalizumab (170 IU/ml), the fact that no differences were found between both subgroups could be due to the size of the samples, since we only had four patients as non‐responders to omalizumab. As it was reported in CSU these patients with cold urticaria not responding to omalizumab showed also a baseline total IgE <40 UI/ml.^11^, ^21^ In addition, and paradoxically, the medians are in the opposite direction to what has been previously seen with CSU, with CSU responders to omalizumab showing higher baseline IgE levels^9^ and high total IgE in CSU representing a high chance of responding to omalizumab treatment.^30^ Therefore, these preliminary results need to be tested in larger samples and are inconclusive regarding the role of baseline IgE serum levels in predicting response to omalizumab in CIndU.

In this vein, although sample sizes for CIndU subtypes do not allow definitive conclusions to be drawn here, exploration of differences by CIndU subtypes shows that, in general, none of the evaluated parameters are affected by urticaria subtype. Therefore, our data suggest that treatment approaches should be similar for different CIndU subtypes.

A final interesting nuance is that we found no differences between subjects who respond completely or partially to omalizumab in any of the evaluated parameters. Thus, it seems that the “true/pure” non‐responders should show an absence of UCT modification and present an idiosyncrasy that makes them show the receptor as a clear biomarker and in a more differential way, without establishing a grade for patients on a continuum according to response level.

### 4.4 Limitations

The current study presents some limitations. Although the current sample size is quite large, this is the first study in this type of patients and it would be interesting to confirm these findings in other, even larger samples, in order to establish statistically powerful comparisons between CIndU subtypes. Therefore, additional multicenter studies are needed to corroborate the current findings.

## 5 CONCLUSION

In summary, the data on basal basophil FcεRI receptor levels and their distribution according to treatment response (to antihistamine and omalizumab) show that patients with CIndU have similar behaviour to patients with CSU patients in terms of baseline total IgE levels, basophil FcεRI receptor and their response to anti‐IgE treatment, without these parameters being affected by induced urticaria subtypes.

## CONFLICT OF INTEREST

Ana Giménez‐Arnau is Medical Advisor for Uriach Pharma, Genentech, Novartis, FAES, GSK, Sanofi–Regeneron, Amgen, Thermo Fisher Scientific, Almirall, LEO‐PHARMA, and Celldex. She has received research grants supported by Uriach Pharma, Novartis, and grants from Instituto Carlos III‐ FEDER/FIS PI17/00198. She performs educational activities for Uriach Pharma, Novartis, Genentech, Menarini, LEO‐PHARMA, GSK, MSD, Almirall, Sanofi and Avene. Clara Ribas‐Llauradó has received a research grant supported Novartis. Gustavo Deza performs educational activities for Novartis. The rest of authors declare no conflict of interest in relation to this work.

## Data Availability

The data that support the findings of this study are available from the corresponding author upon reasonable request.
